# The crosstalk between ubiquitination and GlcNAcylation of CHAF1A regulates HIV-1 latency and reactivation

**DOI:** 10.1128/jvi.01518-25

**Published:** 2025-12-03

**Authors:** Tao Yang, Minghua Chen, Mo Zhou, Xiaohui Deng, Peiming Huang, Siyi Xie, Jianteng Zeng, Jingjing Luo, Yiwen Zhang, Xiancai Ma, Liqin Sun, Jiaye Liu, Hui Zhang, Linghua Li, Bingfeng Liu, Jie Qin, Ting Pan

**Affiliations:** 1 Shenzhen Key Laboratory for Systems Medicine in Inflammatory Diseases, Centre for Infection and Immunity Study (CIIS), School of Medicine, Shenzhen Campus of Sun Yat-Sen University, Sun Yat-Sen University 582261 , Shenzhen, Guangdong, China; 2 Infectious Disease Center, Guangzhou Eighth People’s Hospital, Guangzhou Medical University 26468, Guangzhou, Guangdong, China; 3 Guangdong Institute of Intelligence Science and Technology, Hengqin, Zhuhai, Guangdong, China; 4 Institute of Human Virology, Department of Pathogen Biology and Biosecurity, Key Laboratory of Tropical Disease Control of Ministry Education, Guangdong Engineering Research Center for Antimicrobial Agent and Immunotechnology, Zhongshan School of Medicine, Sun Yat-Sen University 74644 , Guangzhou, Guangdong, China; 5 Guangzhou Laboratory, Guangzhou International Bio-Island, Guangzhou, Guangdong, China; 6 Department of Infectious Diseases, National Clinical Research Center for Infectious Diseases, Shenzhen Third People’s Hospital 638560 , Shenzhen, Guangdong, China; 7 School of Public Health, Shenzhen University Medical School 481870, Shenzhen, Guangdong, China; 8 Department of Gastrointestinal Surgery, The Third People's Hospital of Shenzhen, The Second Hospital Affiliated to Southern University of Science and Technology 255310, Shenzhen, Guangdong, China; University Hospital Tübingen, Tübingen, Germany

**Keywords:** HIV-1 latency, CHAF1A, trifluridine, ubiquitination, O-GlcNAcylation, aging

## Abstract

The persistence of latent HIV-1 reservoirs remains a critical barrier to cure. Current “shock and kill” strategies are limited by ineffective latency-reversing agents (LRAs) and poor understanding of epigenetic regulation. Here, we identify chromatin assembly factor 1 subunit A (CHAF1A), a histone chaperone enforcing HIV-1 latency, as a therapeutic target regulated by antagonistic post-translational modifications: ubiquitination promotes its degradation, while O-GlcNAcylation stabilizes it. We demonstrate that trifluridine, a Food and Drug Administration-approved antiviral drug, reactivates latent HIV-1 by disrupting O-GlcNAcylation, triggering CHAF1A ubiquitination and proteasomal degradation. Notably, CHAF1A expression increases with age in CD4^+^ T cells (>60 years), correlating with deeper proviral reservoirs. This age-dependent accumulation inversely associates with reduced O-GlcNAcase levels, suggesting O-GlcNAcylation-mediated stabilization in aging. Our findings establish CHAF1A as both a therapeutic target and an age-stratifying biomarker, advancing trifluridine as a translatable LRA to enhance reservoir clearance in aging populations—a demographic increasingly impacted by HIV-1 persistence.

**IMPORTANCE:**

HIV-1 latency continues to represent a significant barrier to achieving a cure, particularly in aging populations characterized by expanded viral reservoirs and compromised immune recovery—a challenge further intensified by the absence of therapies specifically designed to target age-related mechanisms. Current latency-reversing agents (LRAs) are insufficient in addressing the metabolic and epigenetic dysregulation that sustains viral persistence in older individuals. In this study, we reveal a dynamic interplay between ubiquitination and O-GlcNAcylation that regulates the stability of CHAF1A, a histone chaperone essential for maintaining HIV-1 latency. We identify trifluridine as a novel LRA capable of disrupting O-GlcNAcylation to degrade CHAF1A, thereby effectively reversing latency in primary cells. This research bridges a critical gap between fundamental virology and clinical gerontology. These findings establish a robust foundation for refining strategies aimed at HIV-1 eradication, with a focus on targeting host metabolic-epigenetic networks to address latency in underserved aging populations.

## INTRODUCTION

Despite antiretroviral therapy (ART), HIV-1 persists in latent reservoirs within memory CD4^+^ T cells, evading immune detection and necessitating lifelong treatment (1, 2). Eradicating these reservoirs remains a critical challenge, as current “shock and kill” strategies are hindered by incomplete latency reversal and off-target toxicity of latency-reversing agents (LRAs) (3). Notably, the clinical management of HIV-1 is increasingly complicated by aging populations, and advances in ART have significantly extended the lifespan of people living with HIV (PLWH). By 2030, approximately 73% of all PLWH will be 50 years or older, with the median age of this population expected to increase progressively over time (4). This demographic shift introduces unique challenges, as older individuals exhibit larger or deeper latent reservoirs, delayed viral rebound post-ART interruption, and diminished immune responses—factors that exacerbate viral persistence and complicate cure strategies (5, 6). This demographic shift underscores the urgent need to identify age-specific biomarkers and therapeutic targets to tailor interventions for aging populations, who remain disproportionately underserved in HIV-1 cure research.

Chromatin assembly factor 1 subunit A (CHAF1A), a histone chaperone critical for heterochromatin formation, has emerged as a key suppressor of HIV-1 transcription (7–9). Our prior work revealed that the CAF-1 complex enforces viral latency through liquid-liquid phase separation (LLPS), forming nuclear condensates at the HIV-1 promoter that recruit repressive epigenetic modifiers (e.g., HDACs and H3K9me3 writers) to stabilize heterochromatin (10). Building on this, we now investigate the post-translational regulation of CHAF1A, focusing on the antagonistic interplay between two modifications: *ubiquitination*, which signals proteasomal degradation, and *O-GlcNAcylation*, a nutrient-sensitive modification that stabilizes chromatin-associated proteins (11, 12). While CHAF1A maintains latency by depositing repressive histone H3 variants, its stability is dynamically regulated by competing post-translational modifications (PTMs). Notably, ubiquitination and O-GlcNAcylation are known to compete for substrate occupancy in other systems (12–15), but their crosstalk in HIV-1 latency remains unexplored. We hypothesize that this PTM axis acts as a molecular switch governing CHAF1A stability, thereby linking cellular metabolism and aging to viral persistence.

The role of PTMs as biomarkers has revolutionized fields such as oncology, where phosphorylation and ubiquitination patterns guide diagnosis, prognosis, and therapeutic targeting (16–19). However, their potential in HIV-1 research—particularly in addressing age-related latency dynamics—remains underexplored. In this study, we investigate how O-GlcNAcylation modulates CHAF1A stability, thereby influencing HIV-1 latency. Using primary CD4^+^ T cell models, we show that trifluridine (TFD), a clinically approved antiviral (20–23), disrupts O-GlcNAc modification, promoting CHAF1A degradation and latent HIV-1 reactivation. Critically, our findings bridge molecular mechanisms to clinical aging challenges: CHAF1A expression increases with age in older individuals (>60 years). Mechanistically, age-dependent declines in O-GlcNAcase (OGA) enhance CHAF1A stabilization via O-GlcNAcylation, linking cellular metabolism and aging to viral persistence. This age-metabolism-latency axis highlights CHAF1A as both a PTM-regulated therapeutic target and a biomarker for stratifying patients by reservoir dynamics, offering a precision framework to address aging-related HIV-1 persistence.

## RESULTS

### Multi-omics analysis reveals ubiquitination and glycosylation jointly participate in CAF-1-related HIV-1 latency

**Fig 1:**
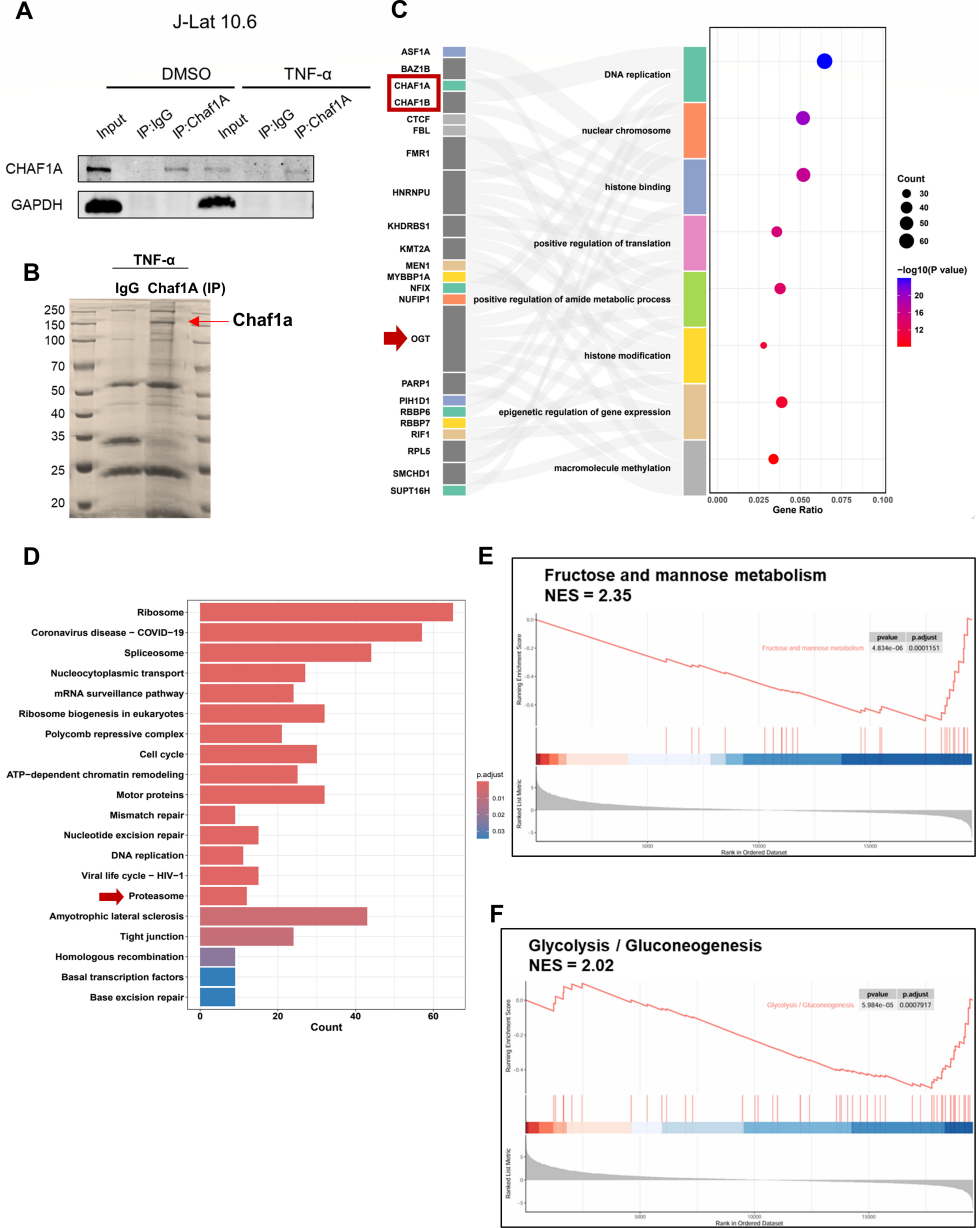
Multi-omics reveals ubiquitin-O-GlcNAc crosstalk in CAF-1-dependent HIV-1 latency. (**A**) J-Lat 10.6 cells were treated with DMSO or TNFα, 24 h later, the cells were lysed and immunoprecipitated with anti-CHAF1A antibody. Western blot was performed with CHAF1A and GAPDH antibodies. (**B**) Silver staining of immunoprecipitation samples following TNF-α activation. (**C**) Combined Sankey and bubble plot showing GO analysis of mass spectrometry-identified ubiquitination/glycosylation-related genes, where the Sankey diagram displays protein-GO term relationships and the bubble plot indicates gene ratios and significance levels (−log10 *P*-value). (**D**) KEGG pathway analysis of mass spectrometry data. (**E and F**) GSEA plots showing the pathways of differentially expressed genes involved in metabolic regulation, including fructose and mannose metabolism and glycolysis/gluconeogenesis.

Building on prior work demonstrating CHAF1A’s role in promoting latency via LLPS at proviral promoters (10), we performed proteomic approaches to dissect its regulatory mechanisms. Using the J-Lat 10.6 latency model, we observed that TNF-α, a canonical LRA, significantly reduced CHAF1A expression levels and its enrichment in J-Lat 10.6 cells, suggesting the presence of a potential regulatory mechanism during viral reactivation (Fig. 1A). To further explore this observation, we performed gel electrophoresis and silver staining on immunoprecipitation samples following TNF-α activation. The results revealed that, compared with the IgG antibody control group, the CHAF1A antibody specifically enriched a number of differentially expressed proteins during the J-Lat 10.6 reactivation (Fig. 1B). Proteomic profiling analysis of endogenous CHAF1A complexes identified several key interaction partners, including its heterodimeric partner CHAF1B and O-GlcNAc transferase (OGT), with significant enrichment of pathways associated with histone binding and epigenetic regulation of gene expression (Fig. 1C). Furthermore, the Kyoto Encyclopedia of Genes and Genomes (KEGG) pathway analysis of the mass spectrometry data demonstrated significant enrichment of proteasomal-mediated degradation and ribosomal-related processes, indicating that both protein turnover and translational regulation may play critical and complementary roles in maintaining HIV latency (Fig. 1D).

Meanwhile, we conducted a systematic analysis of our previous transcriptome data of J-Lat 10.6 cells before and after TNF-α treatment. The results of gene set enrichment analysis (GSEA) indicated that latent cells were in a state of metabolic quiescence, with significant downregulation of fructose/mannose metabolism (NES = −2.11, *P-*adj = 0.002) and glycolysis (NES = −1.72, *P-*adj = 0.009) pathways, which was highly consistent with the nutrient-restricted state of quiescent cells (Fig. 1E and F). Additionally, this result was in line with the trend of CHAF1A expression level changes, suggesting that CHAF1A might play a stabilizing role in maintaining HIV latency by integrating epigenetic repression with metabolic dormancy. More importantly, integration of mass spectrometry analysis with transcriptomic data revealed the co-enrichment of OGT and proteasome-related pathways, suggesting that CHAF1A may serve as a central node at the intersection of two opposing PTM pathways: O-GlcNAc glycosylation, which stabilizes chromatin-associated proteins, and ubiquitination, which targets these proteins for degradation. Our multi-omics investigation of CHAF1A-mediated HIV-1 latency revealed critical insights into the crosstalk between PTMs that enforce HIV-1 latency, providing a mechanistic foundation for targeting the interplay between ubiquitination and glycosylation to disrupt viral persistence.

### OGT/OGA regulates CHAF1A stability via antagonistic ubiquitination-O-GlcNAcylation crosstalk

**Fig 2:**
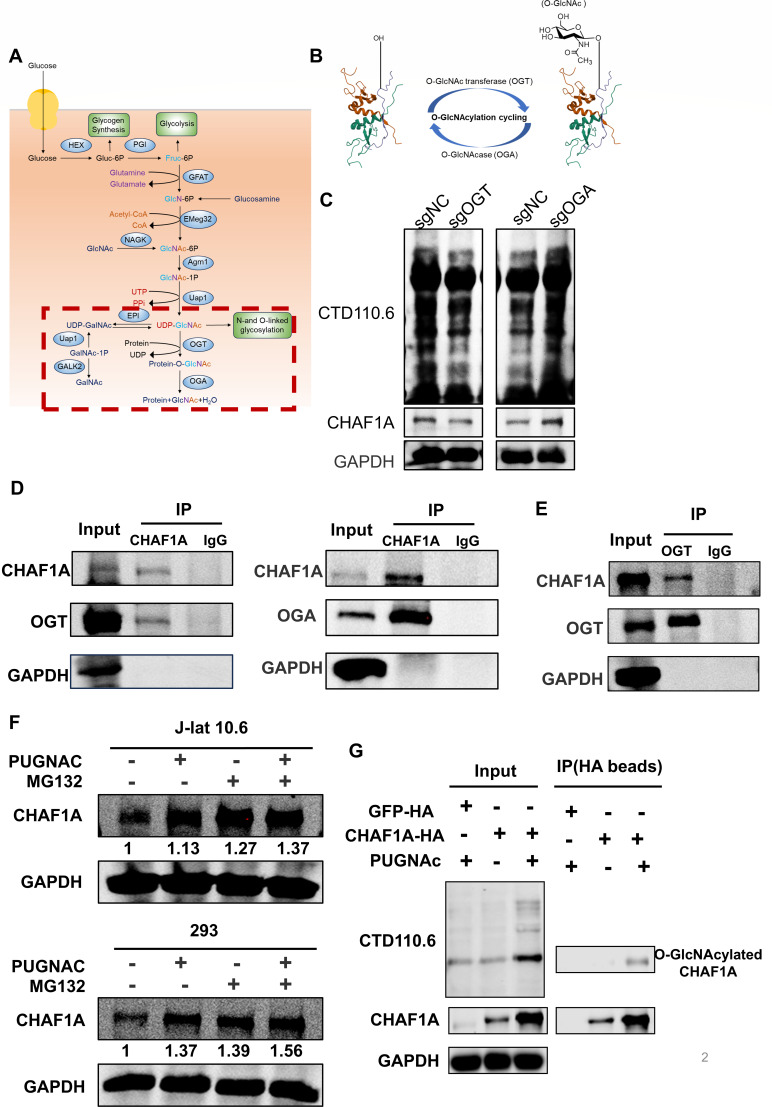
OGT/OGA balance CHAF1A stability via ubiquitin-O-GlcNAc antagonism. (**A**) Illustration of the HBP and O-GlcNAcylation mechanism. (**B**) Dynamic regulation of O-GlcNAcylation by OGT and OGA enzymes. (**C**) J-Lat 10.6 cells were infected with sgRNA targeting OGT (sgOGT) or OGA (sgOGA), with scrambled sgRNA as control (sgNC). Cells were lysed and analyzed by western blot using CTD110.6 (O-GlcNAc), anti-CHAF1A, and anti-GAPDH antibodies. (**D**) CHAF1A interacts with both OGT and OGA in Jutkat cells. Jurkat cells were harvested, and cell lysates were immunoprecipitated with anti-CHAF1A antibody or IgG control, followed by western blot analysis. GAPDH served as loading control for input samples. (**E**) Endogenous OGT and OGA interact with CHAF1A in Jurkat cells. Jurkat cells were harvested and cell lysates were immunoprecipitated with anti-OGT antibody, with IgG as control. GAPDH served as loading control for input samples. (**F**) Western blot analysis for ubiquitin on CHAF1A immunoprecipitated from J-Lat 10.6 and HEK293T cells in the presence/absence of OGA inhibitor PUGNAc. The cells were treated with/without MG132 for proteasome inhibition. Cells were treated with 50 µM PUGNAC (an inhibitor of OGA) for 48 h, followed by an additional 6-h treatment with 20 µM MG132 prior to harvest. (**G**) HEK293T cells transfected with HA-tagged CHAF1A or GFP, and then treated with PUGNAc or not. The O-GlcNAcylated CHAF1A ubiquitination was analyzed by Co-IP with anti-HA beads followed by western blot with CTD110.6. GAPDH was shown as a loading control.

The interplay between ubiquitination and O-GlcNAcylation can dynamically regulate protein stability (24, 25). Here, we investigated this crosstalk in the context of CHAF1A, focusing on the opposing roles of OGT and OGA. In the glycolytic pathway, ~2%–5% of glucose flux is diverted through the hexosamine biosynthesis pathway (HBP) to generate UDP-N-acetylglucosamine (UDP-GlcNAc), the obligate donor substrate for O-GlcNAcylation(26) (Fig. 2A). However, unlike other modifications, the addition and removal of this modification is performed with a single set of enzymes (27, 28). O-GlcNAc glycosylation is catalyzed by OGT, while de-glycosylation is catalyzed by OGA (Fig. 2B). To investigate the impact of O-GlcNAcylation on CHAF1A stability, we genetically perturbed the O-GlcNAc cycling enzymes. Specifically, we used siRNA to knock down OGT and OGA in 293T cells and single-guide RNA (sgRNA) in J-Lat 10.6 cells. Our experiments revealed that OGT knockdown consistently decreased overall cellular O-GlcNAcylation and consequently reduced CHAF1A expression. Conversely, OGA knockdown led to increased O-GlcNAcylation and elevated CHAF1A levels (Fig. 2C; Fig. S1). These results strongly suggest that the OGT/OGA enzymatic pair dynamically regulates CHAF1A protein levels via O-GlcNAc modification.

To further validate the direct association between CHAF1A and these key enzymes, we performed co-immunoprecipitation (Co-IP) assays. Endogenous CHAF1A was found to interact directly with both OGT and OGA (Fig. 2D). Reciprocal Co-IP experiments further confirmed that endogenous OGT specifically complexed with CHAF1A (Fig. 2E). These findings firmly establish that CHAF1A forms protein complexes with both OGT and OGA, thereby positioning these enzymes as critical mediators of CHAF1A stability through O-GlcNAc modification. Conversely, pharmacological inhibition of OGA with PUGNAc enhanced overall O-GlcNAcylation and led to a noticeable increase in CHAF1A protein levels (Fig. 2G). This pharmacological evidence further corroborates that O-GlcNAcylation stabilizes CHAF1A, likely by competitively interfering with its ubiquitination.

Collectively, these findings demonstrate that OGT/OGA-mediated O-GlcNAcylation antagonistically regulates CHAF1A stability by counteracting ubiquitination. This antagonistic PTM crosstalk provides a direct molecular link between the cellular metabolic state and the maintenance of HIV-1 latency.

### Targeted screening identifies TFD as a CHAF1A degrader via ubiquitination-glycosylation crosstalk

**Fig 3:**
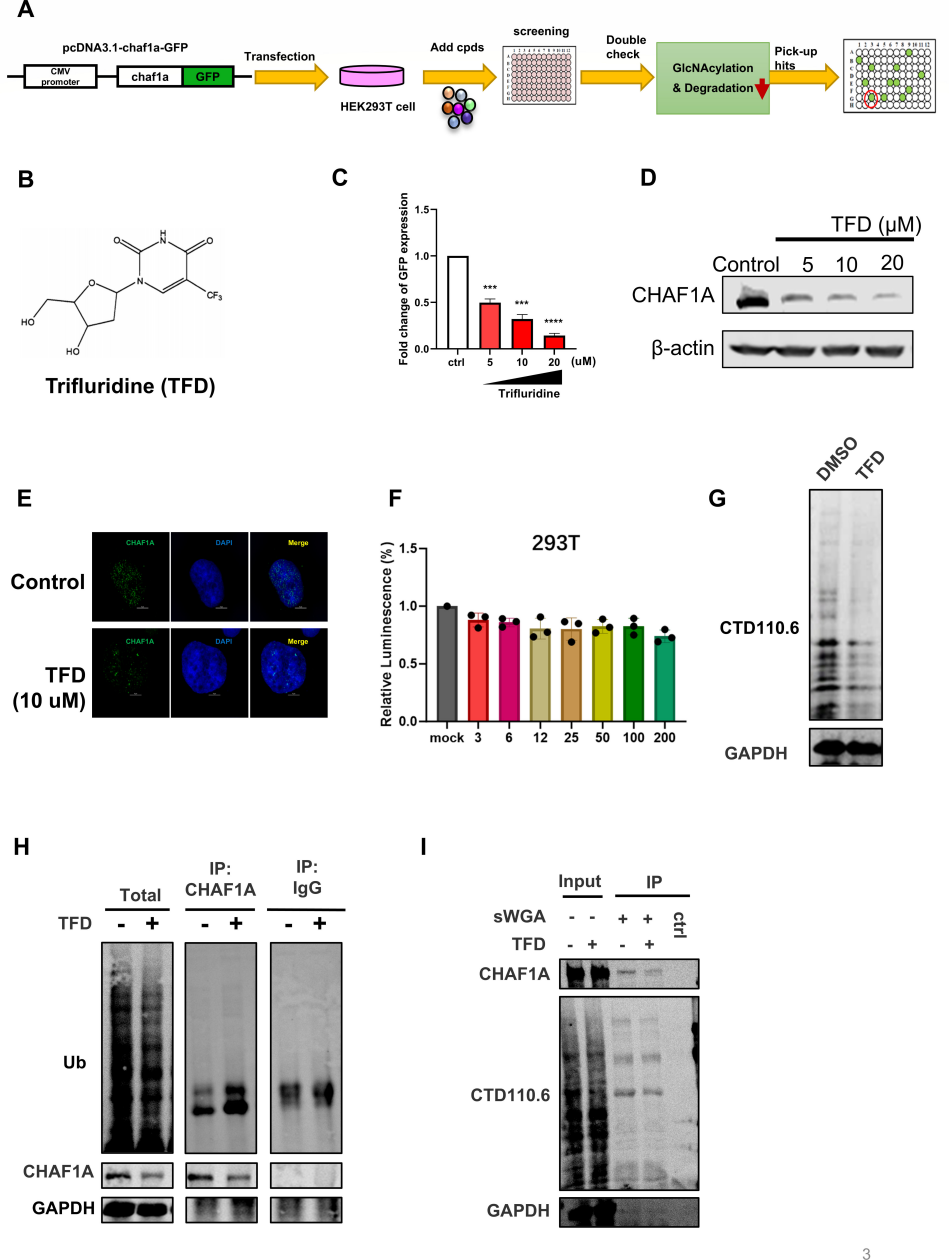
TFD targets CHAF1A through ubiquitin-O-GlcNAc interplay. (**A**) Schematic representation of the high-throughput sequencing method. (**B**) Chemical structure of TFD. (**C and D**) Degradation efficiency of TFD to CHAF1A in HEK293T cells at the indicated concentrations. (**E**) HEK293T cells were treated with TFD at 10 µM for 48 h, followed by fixed and immunofluorescence. (**F**) Cell viability was assessed using CellTiter-Glo assay after 48-h treatment with indicated concentrations of TFD. HEK293T cells maintained >80% viability at all concentrations. Data represent mean ± SEM from three independent experiments. (**G**) J-lat10.6 cells treated with PBS or 10 µM TFD for 48 h were harvested. The level of O-GlcNAc was determined in total cell lysates by western blotting with CTD110.6 antibody. (**H**) J-Lat 10.6 cells were treated with 10 µM TFD and MG132 (20 µM, 6 h) for 48 h. Immunoprecipitation with anti-CHAF1A antibody followed by Western blot with ubiquitin antibody. (**I**) Jurkat cells were lysed with or without 10 µM TFD treatment, and O-GlcNAc-modified proteins were enriched by sWGA. Input and sWGA pull-down (IP) fractions were analyzed by immunoblotting using CTD110.6 (O-GlcNAc), anti-CHAF1A, and GAPDH antibodies.

To identify compounds that specifically degrade CHAF1A, we screened the US DRUG COLLECTION library using a green fluorescent protein (GFP)-CHAF1A fusion protein as an indicator. We constructed a pcDNA3.1-CFP plasmid as an internal reference to transfection control (Fig. 3A). After three rounds of screening, TFD was identified as a candidate drug that significantly degraded CHAF1A (Fig. S2; Fig. 3B). Further verification through GFP fluorescence experiments and western blot experiments confirmed that TFD reduced both exogenous GFP-CHAF1A and endogenous CHAF1A in a dose-dependent manner (Fig. 3C and D). Immunofluorescence further demonstrated diminished nuclear CHAF1A expression upon TFD treatment (Fig. 3E; Fig. S3), consistent with proteasomal degradation. Cytotoxicity profiling revealed that TFD maintained >90% cell viability in primary human peripheral blood mononuclear cells (PBMCs) and cell lines (HEK293T and TZM-bl) at concentrations up to 200 µM, which is sufficient for CHAF1A degradation (Fig. 3F; Fig. S4A and B). However, we observed higher sensitivity in certain lymphocyte cell lines (Jurkat and J-Lat 10.6) at the highest doses up to 10 µM (Fig. S4C and D). Notably, while TFD is a nucleoside analog that can affect both host and viral DNA synthesis, its safety and tolerability have been established in clinical settings when administered appropriately in combination therapies for cancer, such as TFD/tipiracil plus bevacizumab for refractory metastatic colorectal cancer (29) and TFD/tipiracil in advanced gastric or colorectal cancers (30, 31). These clinical data indicate that, when properly dosed and formulated, TFD has the potential for systemic administration.

To investigate the underlying mechanism, we initially treated human T lymphocyte cell line Jurkat T cells with TFD. The results demonstrated that TFD significantly reduced global cellular glycosylation levels (Fig. 3G). This effect was similarly observed in human primary CD4^+^ T cells (Fig. S5). Further endogenous co-IP experiments using a CHAF1A-specific antibody revealed that TFD treatment markedly increased CHAF1A ubiquitination, suggesting the involvement of the ubiquitin-proteasome system in its degradation (Fig. 3H). Moreover, sWAG analysis confirmed that CHAF1A glycosylation was substantially diminished under TFD treatment conditions (Fig. 3I), indicating that TFD facilitated CHAF1A ubiquitination and subsequent degradation by disrupting O-GlcNAc-mediated stabilization.

These results position TFD as a dual-action compound that destabilizes CHAF1A by simultaneously suppressing O-GlcNAcylation and promoting ubiquitination. This unique mechanism highlights its potential as a precision LRA tailored to aging-associated HIV-1 reservoirs.

### The E3 ubiquitin ligase MIB1 mediates TFD-induced CHAF1A ubiquitination and degradation

**Fig 4:**
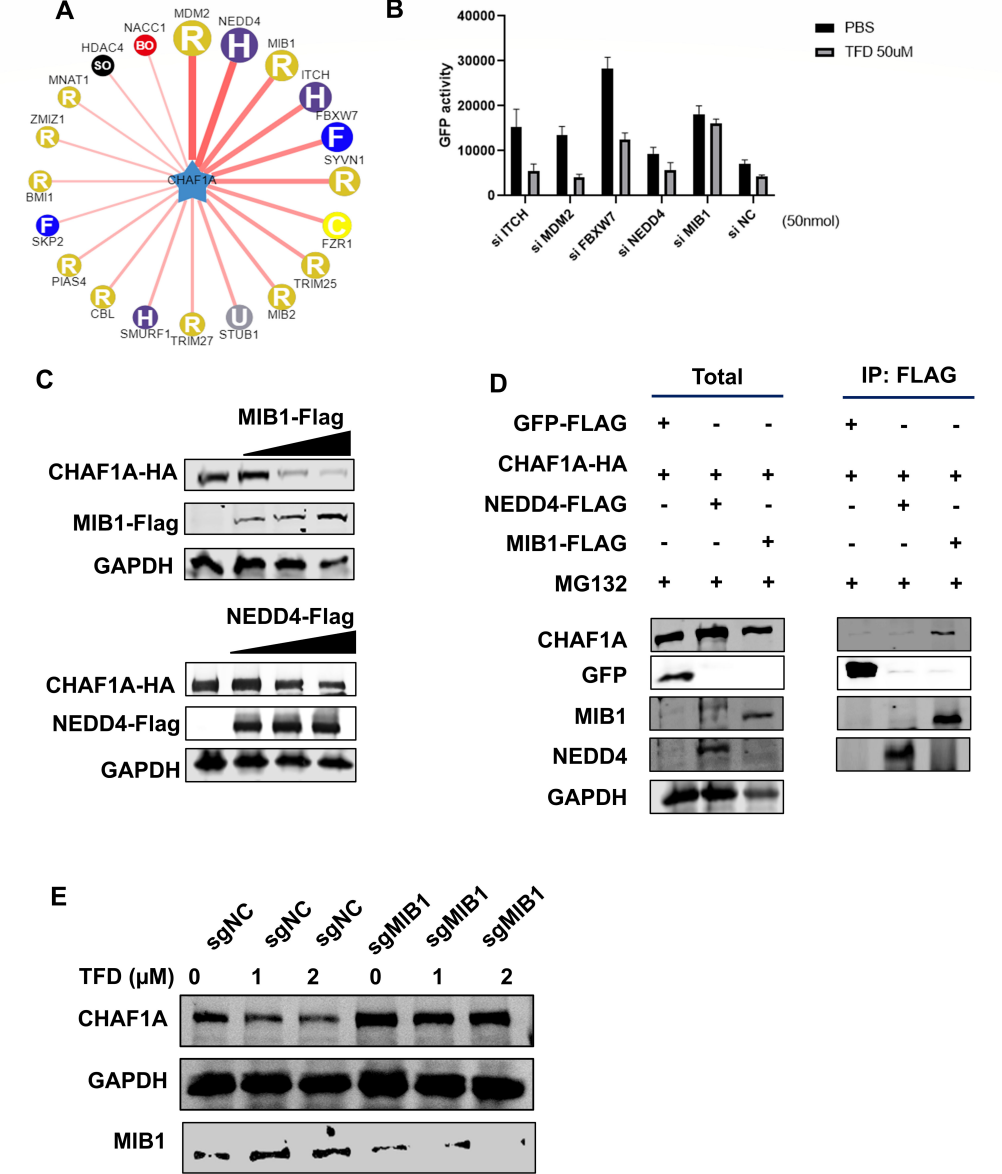
E3 ligase MIB1 triggers CHAF1A ubiquitination by TFD. (**A**) E3 ligases predicted by the UbiBrowser database. (**B**) HEK293T cells were co-transfected with co-transfected with pEGFP-CHAF1A-C1, pcDNA3.1-CFP, and siRNAs (siNC [control], siITCH, siMDM2, siFBXW7, siNEDD4, and siMIB1), then treated with TFD. GFP fluorescence intensity is used for quantitative drug degradation. (**C**) HEK293T cells were co-transfected with CHAF1A-HA and increased amounts of MIB1-Flag (up) or NEDD4-Flag (down). At 24 h post-infection, the cells were lysed, and western blot was performed with HA, FLAG, and GAPDH antibodies. (**D**) HEK293T cells were co-transfected with CHAF1A-HA and GFP-Flag (control), NEDD4-Flag, or MIB1-Flag. Then the cells were treated with MG132 for proteasome inhibition. At 48 h post-infection, cells were lysed, and western blot was performed with HA, FLAG, and GAPDH antibodies. (**E**) J-Lat10.6 cells with MIB1 knockout (sgMIB1) or control sgRNA (sgNC) were treated with the indicated concentrations of TFD for 24 h. CHAF1A protein levels were analyzed by western blot, with GAPDH as a loading control.

E3 ubiquitin ligases are essential for substrate recognition and ubiquitin transfer in protein degradation (32). To define the role of E3 ligase in TFD-mediated CHAF1A ubiquitination, we initially used the UbiBrowser database (UbiBrowser [bio-it.cn]) and obtained several predicted proteins (Fig. 4A). Among the top 5 candidates, siRNA screening revealed that MIB1 (a RING-domain E3 ligase) and NEDD4 (a HECT-domain E3 ligase) partially reversed TFD-induced CHAF1A degradation (Fig. 4B) (33, 34). While NEDD4 knockdown modestly attenuated CHAF1A protein levels, MIB1 silencing nearly abolished it, suggesting MIB1 plays a dominant role.

To validate these candidates, we overexpressed MIB1 or NEDD4 in HEK293T cells. Both ligases promoted CHAF1A degradation in a dose-dependent manner (Fig. 4C). However, Co-IP assays demonstrated a direct interaction between CHAF1A and MIB1, but not NEDD4 (Fig. 4D), indicating that NEDD4’s effect may occur indirectly through proteasomal regulation. To definitively establish the requirement of MIB1 in TFD-induced CHAF1A degradation, we generated MIB1 knockout (sgMIB1) J-Lat 10.6 cells using CRISPR/Cas9 and subsequently assessed CHAF1A protein levels following TFD treatment. In control cells (sgRNA as control [sgNC]), TFD induced a clear dose-dependent reduction of CHAF1A. Strikingly, MIB1 knockout largely abolished this effect, with CHAF1A protein levels remaining stable even at higher TFD concentrations (Fig. 4E). These results conclusively confirm that MIB1 is essential for TFD-triggered CHAF1A degradation in J-Lat 10.6 cells.

By integrating loss-of-function, gain-of-function, and direct interaction analyses, we conclusively identify MIB1 as the E3 ubiquitin ligase responsible for TFD-driven CHAF1A ubiquitination and subsequent degradation. This discovery positions MIB1 as a critical node in the PTM crosstalk regulating HIV-1 latency and highlights its therapeutic potential for targeted reservoir clearance.

### Inhibiting OGT or overexpressing OGA reactivates latent HIV-1 by destabilizing CHAF1A

**Fig 5:**
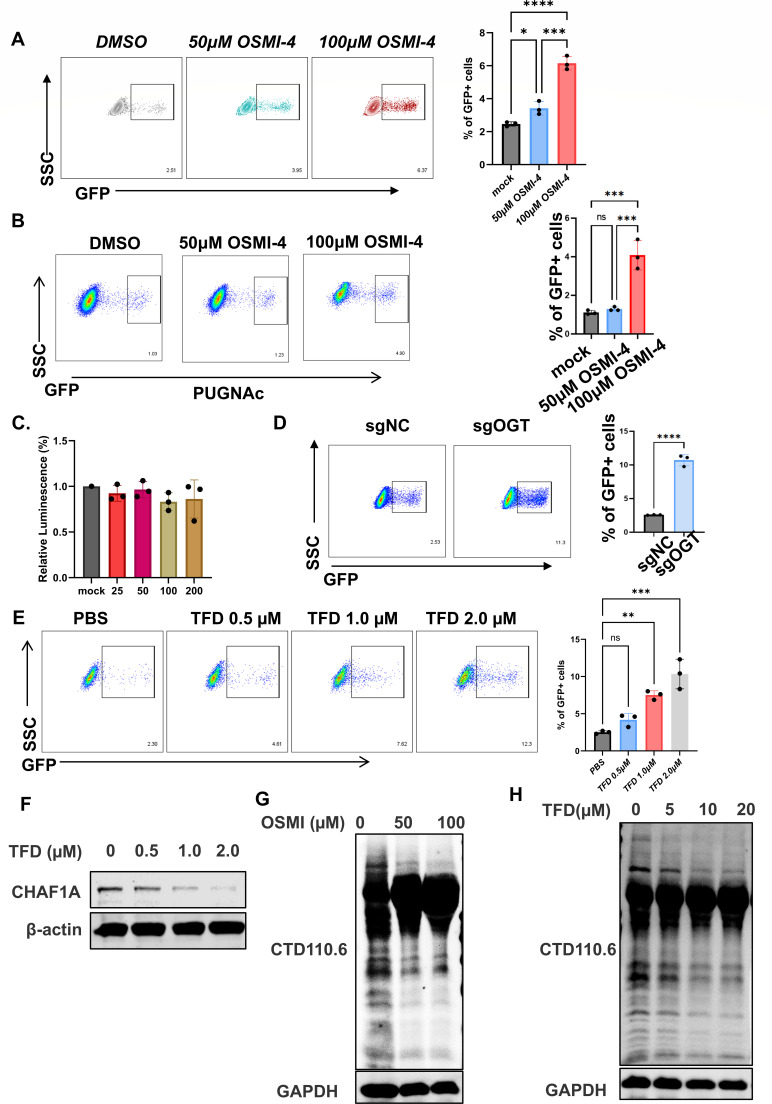
Targeting OGT/OGA destabilizes CHAF1A to reactivate latent HIV-1. (**A**) J-Lat 10.6 cells were treated with OSMI-4 (an inhibitor of OGT), DMSO was used as a control. 48 h after treatment, the cells were harvested, and the percentage of GFP-positive cells was measured by flow cytometry. (**B**) J-Lat 10.6 cells were pre-treated with OGA inhibitor PUGNAc for 24 h, followed by OGT inhibitor OSMI-4 at indicated concentrations for 48h. HIV-1 reactivation was measured by the percentage of GFP-positive cells using flow cytometry. (**C **)Cell viability was evaluated by CellTiter-Glo assay after 48-h treatment with different concentrations of OSMI-4 (25, 50, 100, and 200 µM). Data are presented as mean ± SD from three independent experiments. Statistical significance was determined by one-way ANOVA. (**D**) J-Lat 10.6 cells were transduced with sgRNA targeting OGT (sgOGT) or a non-targeting control (sgNC). After selection, HIV-1 reactivation was determined by flow cytometry for GFP expression. (**E and F**) J-Lat 10.6 cells were treated with increased concentration of TFD, PBS was used as a control. 24 h after treatment, the cells were harvested, the percentage of GFP-positive cells was measured by flow cytometry (**E**), and the cells were lysed, and western blot was performed with CHAF1A and β-actin antibodies (**F**). (**G**) OGT inhibitor reduces global O-GlcNAcylation in J-Lat 10.6 cells. J-Lat 10.6 cells were treated with OGT inhibitor at 0, 50, or 100 µM for 48 h. Whole cell lysates were analyzed by western blot using an anti-O-GlcNAc antibody to assess global protein O-GlcNAcylation. GAPDH was used as a loading control. (**H**) TFD treatment reduces global O-GlcNAcylation levels in a dose-dependent manner. J-Lat 10.6 cells were treated with increasing concentrations of TFD (0, 5, 10, and 20 µM) for 48 h. Whole cell lysates were analyzed by western blot using anti-O-GlcNAc antibody (CTD110.6) to assess global protein O-GlcNAcylation. GAPDH was used as a loading control. ns, not significant (*P* > 0.05); *P < 0.05; **P < 0.01; ***P < 0.001; ****P < 0.0001.

To determine whether O-GlcNAcylation directly modulates HIV-1 latency, we treated J-Lat 10.6 cells with OSMI-4, a selective OGT inhibitor (35). OSMI-4 significantly reduced global O-GlcNAcylation and reactivated latent HIV-1 (Fig. 5A), thereby demonstrating that suppressing glycosylation disrupts viral quiescence. Furthermore, pre-treatment with the OGA inhibitor PUGNAc markedly reduced OSMI-4-induced HIV-1 reactivation, suggesting that O-GlcNAcylation directly regulates HIV-1 latency (Fig. 5B). Cell viability assays using CellTiter-Glo confirmed that OSMI-4 did not induce significant cytotoxicity at concentrations up to 200 µM (Fig. 5C), ruling out non-specific toxic effects. Similarly, genetic deletion of OGT, the enzyme responsible for O-GlcNAc transfer, also enhanced viral reactivation (Fig. 5D), providing further genetic evidence that reduced O-GlcNAcylation destabilizes latency. Importantly, previous work has demonstrated that knockout of CHAF1A effectively reactivates latent HIV-1 in J-Lat 10.6 cells, highlighting the critical role of CHAF1A in maintaining latency (10).

We next validated the latency-reversing activity of TFD. TFD reactivated latent HIV-1 in J-Lat 10.6 cells in a concentration-dependent manner (Fig. 5E), paralleling its ability to degrade endogenous CHAF1A (Fig. 5F). Meanwhile, western blot analysis following OSMI treatment revealed a dose-dependent decrease in global O-GlcNAc levels (Fig. 5G), whereas TFD treatment resulted in a dose-dependent increase in global O-GlcNAc levels (Fig. 5H). This dual effect—suppressing O-GlcNAcylation while promoting CHAF1A ubiquitination—positions TFD as a mechanistically unique LRA.

Given CHAF1A’s initial identification in TNF-α-stimulated Jurkat T cells, we conducted TNF-α stimulation and receptor blockade experiments to validate our proposed mechanism and link it to prior findings. In J-Lat 10.6 cells, combined TFD and TNF-α treatment synergistically enhanced HIV-1 activation. Importantly, while the TNF-α receptor blocker Atrosab significantly inhibited TNF-α-mediated activation, it had no appreciable effect on TFD-induced latency reversal (Fig. S6A and B). Furthermore, RNA-seq analysis of TFD-treated cells revealed no significant changes in the expression of key HIV latency regulators such as BRD4 and NF-κB (Fig. S6C), indicating that TFD acts independently of these established pathways. In parallel, CHAF1A rescue experiments in TZM-BL cells showed that CHAF1A overexpression completely reversed trifluorothymidine-induced viral reactivation (Fig. S6D), thereby confirming its role as a critical downstream effector. Collectively, the strong correlation among O-GlcNAc modification, CHAF1A dynamics, and HIV reactivation, together with the rescue data, supports the existence of a dominant, specific signaling pathway distinct from non-specific toxic effects. These findings establish that targeting the O-GlcNAcylation-ubiquitination axis of CHAF1A effectively disrupts HIV-1 latency.

### TFD reactivates latent HIV-1 in primary cells, and CHAF1A expression correlates with aging

**Fig 6:**
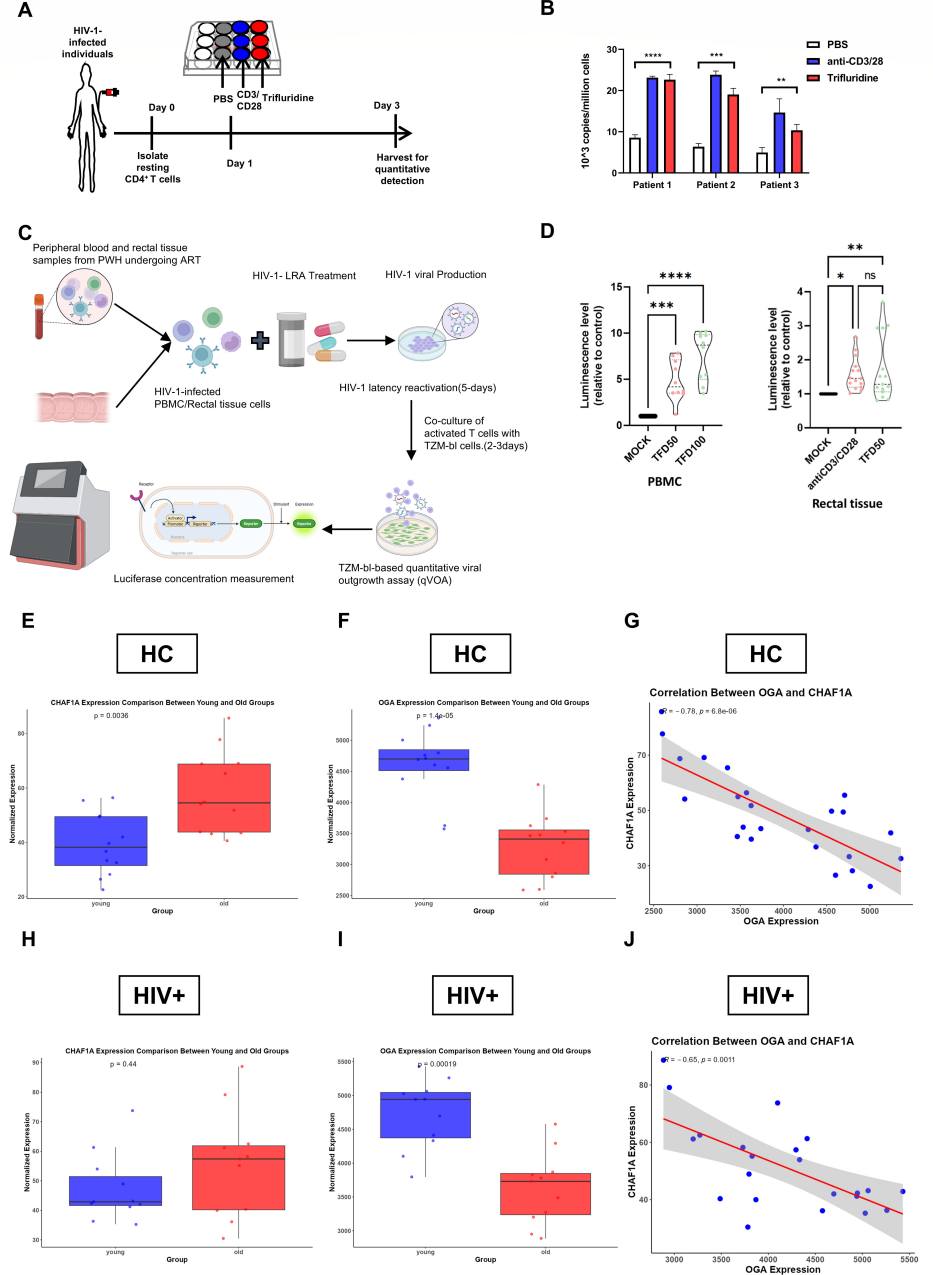
TFD reactivates latent HIV-1 and links CHAF1A to aging. (**A**) The procedure of HIV-1 reactivation in primary CD4^+^ T cells of PLWH. (**B**) Resting CD4^+^ T cells were isolated from HIV-1-infected individuals; 1 day later, the cells were treated with PBS (control), anti-CD3/CD28, or TFD. The cells were harvested for HIV-1 RNA quantitative detection 2 days after treatment. The corresponding statistical results were presented with three individual donors. (**C**) Schematic overview of the TZM-bl viral outgrowth assay (qVOA) using peripheral blood and rectal tissue samples from people with HIV (PWH) undergoing ART. (**D**) Quantification of HIV-1 reactivation by luciferase assay in samples from five donors. Cells were treated with DMSO (MOCK), anti-CD3/CD28, or TFD at 50 µM (TFD50) or 100 µM (TFD100). Data are shown as mean ± SD; statistical significance was determined by two-way ANOVA with Tukey’s multiple comparison test. (**E–G**) Analysis in healthy controls (HC): (**E**) CHAF1A expression is significantly increased in the old group; (**F**) OGA expression is significantly decreased in the old group; (**G**) strong negative correlation between CHAF1A and OGA expression. (**H–J**) Analysis in HIV+ individuals: (**H**) CHAF1A expression shows an increasing trend in the old group; (**I**) OGA expression is significantly decreased in the old group; (**J**) moderate negative correlation between CHAF1A and OGA expressions. ns, not significant (*P* > 0.05); *P < 0.05; **P < 0.01; ***P < 0.001; ****P < 0.0001.

To validate TFD as a clinically relevant LRA, we tested its efficacy in primary CD4^+^ T cells isolated from three HIV-1-infected individuals on suppressive combination ART (cART) for >6 months (Fig. 6A). TFD significantly upregulated intracellular HIV-1 RNA, achieving reactivation levels comparable to the potent αCD3/αCD28 stimulation (Fig. 6B). This demonstrates TFD’s ability to target latent reservoirs in patient-derived cells, a critical milestone for translational relevance. To further assess the potential of TFD as a LRA, we tested its ability to reactivate latent HIV-1 in *ex vivo* primary cells from ART-treated individuals using the TZM-bl-based qVOA assay (36, 37). PBMCs and rectal cells were treated with TFD or anti-CD3/CD28 antibodies, and viral outgrowth was measured by luciferase activity (Fig. 6C). TFD significantly reactivated HIV-1 in both blood and rectal samples (Fig. 6D). This finding strongly supports the feasibility of exploring TFD as a localized therapeutic strategy for targeting tissue-resident reservoirs, leveraging its existing safety profile and approved route of administration for a related viral indication.

We further investigated the age-dependent expression of CHAF1A and its regulatory enzyme, OGA (MGEA5), using a public data set (GSE178670) from both healthy and HIV-positive individuals (6). In healthy controls, CHAF1A expression was significantly higher in older individuals (over 60 years) than in younger counterparts (*P* = 0.0036; Fig. 6E). Conversely, OGA levels significantly declined with age (*P* = 1.4 × 10⁻⁵; Fig. 6F). These changes showed a strong inverse correlation (*r* = −0.78, *P* = 6.8 × 10⁻⁶; Fig. 6G), suggesting that age-related OGA loss drives CHAF1A accumulation. In HIV-positive individuals, OGA levels also declined sharply in older donors (*P* = 0.00019; Fig. 6H), and the CHAF1A-OGA correlation remained negative (*r* = −0.65, *P* = 0.0011; Fig. 6I). While the trend for CHAF1A to increase with age was not statistically significant in this cohort (*P* = 0.44; Fig. 6J), these findings suggest that chronic HIV infection or cART may modulate CHAF1A dynamics, but the core OGA-CHAF1A axis persists.

These results position CHAF1A as a potential age-associated latency stabilizer and underscore TFD’s ability to counteract its accumulation in older individuals. Our findings highlight the need for age-stratified HIV-1 cure strategies.

## DISCUSSION

**Fig 7:**
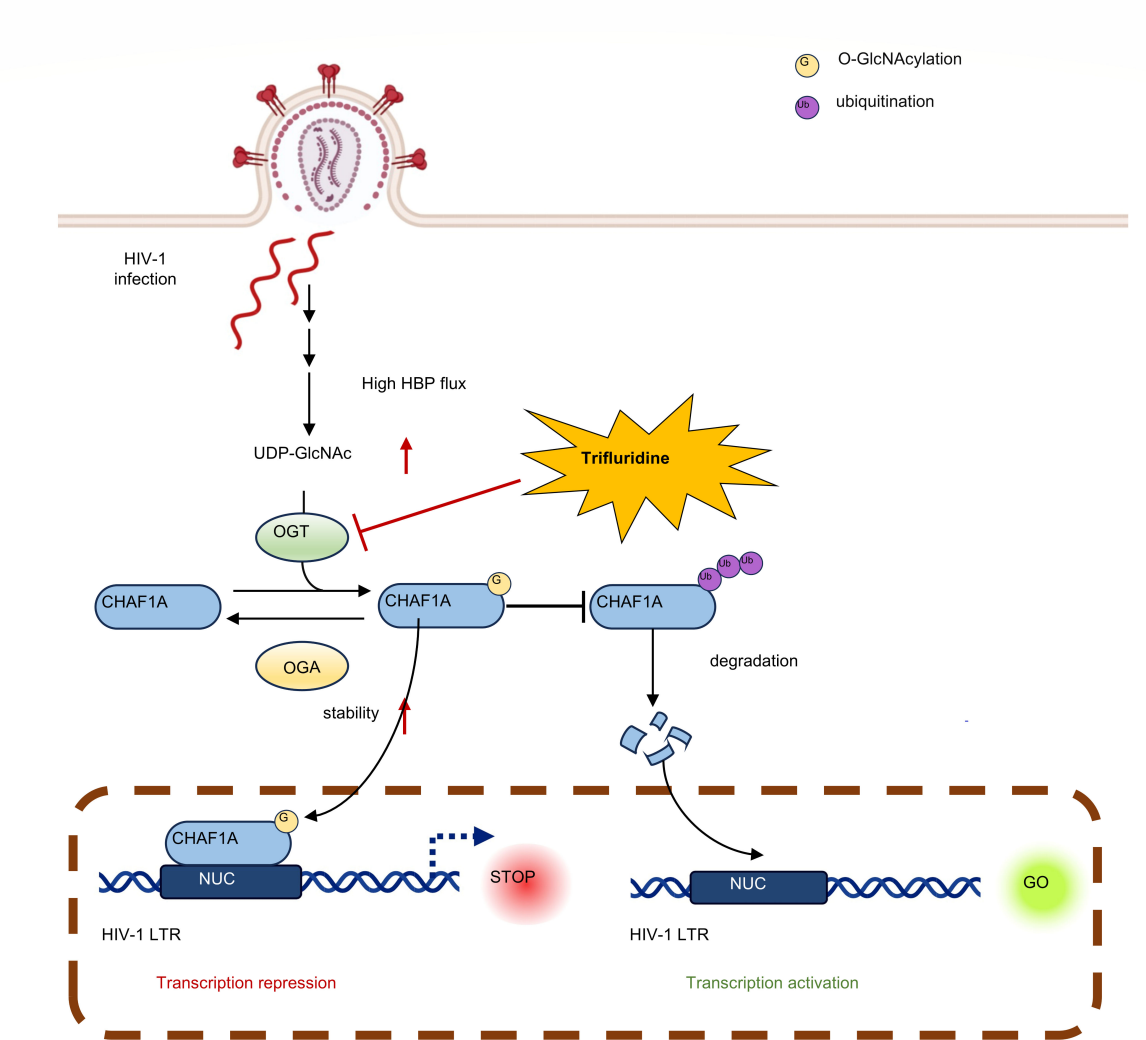
Schematic representation of CHAF1A-mediated HIV-1 latency and reactivation. Aberrant glucose metabolism in HIV-infected cells leads to the accumulation of UDP-GlcNAc, which participates in the O-GlcNAcylation of CHAF1A, thereby stabilizing its expression. This stabilization promotes HIV-1 latency and the establishment of deep viral reservoirs. Exogenous administration of TFD removes the O-GlcNAc modification from CHAF1A and mediates its degradation via the E3 ubiquitin ligase MIB1, thereby releasing the suppression on the HIV-1 genome and reactivating the latent viral reservoir.

Our findings demonstrate that O-GlcNAcylation stabilizes CHAF1A by competitively blocking ubiquitination, thereby reinforcing heterochromatin at latent proviruses (Fig. 7). Conversely, disrupting O-GlcNAcylation—via TFD or enzymatic modulation of OGT/OGA—shifts the balance toward ubiquitin-dependent CHAF1A degradation, derepressing viral transcription (33). This PTM crosstalk not only elucidates a nutrient-sensitive “molecular switch” controlling latency but also positions CHAF1A as a dynamic therapeutic node amenable to pharmacological intervention. The discovery of CHAF1A as a central regulator of HIV-1 latency, governed by the antagonistic interplay between ubiquitination and O-GlcNAcylation, provides a paradigm-shifting framework for understanding viral persistence.

TFD, a clinically approved antiviral drug, emerges as a compelling LRA by uniquely targeting CHAF1A’s PTM equilibrium. Unlike broad-spectrum histone deacetylase inhibitors (e.g., vorinostat) or non-specific PKC agonists, TFD’s specificity for O-GlcNAcylation pathways minimizes off-target toxicity—a critical limitation of current LRAs (3, 38). By destabilizing CHAF1A, TFD selectively reactivates latent HIV-1 without global chromatin disruption, offering a safer profile for clinical translation (39). The identification of MIB1 as the E3 ligase mediating TFD-induced CHAF1A ubiquitination further validates its mechanism, providing a roadmap for optimizing small-molecule inhibitors targeting this axis (40, 41). TFD functions as a novel LRA with dual mechanisms.

Our study also establishes TFD as a promising LRA with a clearly defined therapeutic window. Through rigorous cytotoxicity assessments using the CellTiter-Glo assay, we found that TFD can effectively reactivate latent HIV-1 at concentrations that do not cause significant toxicity in multiple cell lines and primary cells. This finding is crucial, as it addresses concerns about TFD’s known systemic toxicity. Furthermore, we propose two distinct therapeutic strategies that leverage TFD’s established clinical use and our new data. First, clinical literature shows that TFD is already safely used in systemic cancer treatment regimens, where appropriate dosing and scheduling successfully mitigate toxicity (29–31, 42). This supports the feasibility of controlled systemic administration for HIV cure strategies. Second, given the drug’s approved topical use for HSV, we investigated its potential for localized HIV-1 reservoir targeting. Our data from *ex vivo* rectal tissue explants demonstrated that TFD robustly reactivates latent virus in this relevant tissue compartment. This suggests a viable localized therapeutic strategy for tissue-resident reservoirs, which takes advantage of the drug’s existing safety profile and approved route of administration for a related viral indication.

Moreover, our identification of CHAF1A as an age-related biomarker addresses a pressing clinical challenge: older individuals (≥60 years) exhibit larger HIV-1 reservoirs and delayed viral rebound post-ART, complicating cure strategies (43). The inverse correlation between CHAF1A and OGA levels in aging CD4^+^ T cells suggests that age-associated declines in OGA activity enhance O-GlcNAcylation-driven CHAF1A stabilization, perpetuating latency. This finding underscores the need for age-stratified approaches to reservoir clearance. For instance, TFD’s efficacy may be heightened in older patients with elevated CHAF1A, while younger cohorts might benefit from combined OGT inhibition and immune checkpoint therapies. The finding of age-dependent CHAF1A regulation may be an implication for HIV-1 precision medicine.

While our study establishes CHAF1A’s PTM crosstalk as a key latency mechanism, several questions remain. First, the PTM of O-GlcNAcylation is nutrient-sensitive, which may link cellular metabolic state to HIV-1 latency and suggest metabolic interventions (e.g., modulating glucose/glutamine availability) could enhance LRA efficacy. Second, the discovery of MIB1 as a CHAF1A-specific E3 ligase opens avenues for developing proteolysis-targeting chimeras to enhance degradation efficiency. Finally, the tissue-specificity of CHAF1A regulation in sanctuary sites (e.g., central nervous system and lymphoid tissues) warrants exploration and long-term safety of TFD in HIV-1 patients—particularly its impact on host chromatin stability—requires validation.

In conclusion, our work not only uncovers a novel mechanism of HIV latency reversal but also provides a compelling rationale for further exploring TFD as an LRA, especially for targeting tissue-based reservoirs. By unraveling the PTM crosstalk governing CHAF1A, this work redefines HIV-1 latency as a metabolically plastic state amenable to precision modulation. TFD’s repurposing as a CHAF1A-targeted LRA, coupled with CHAF1A’s potential as an age-related biomarker, bridges molecular discovery to critical clinical needs. As the global population of PLWH-1 ages, our findings advocate for tailored strategies that integrate metabolic regulation, aging biology, and targeted latency reversal—a triad essential for achieving functional cures.

## MATERIALS AND METHODS

### Study approval

The CD4^+^ T cells from healthy adult donors were provided by the Institutional Review Board of Guangzhou Blood Center (Guangzhou, Guangdong, China). The HIV-1-infected donors, who had undergone cART with undetectable HIV-1 viral loads in plasma (fewer than 50 copies/ml) for more than 6 months, were recruited for our study by the Department of Infectious Diseases in Guangzhou 8th People’s Hospital, Guangzhou, China. All donors provided written informed consent.

### Cell culture

HEK293T (CVCL_0063; ATCC), TZM-bl (8129) cells obtained from NIH AIDS Reagent Program, were incubated in Dulbecco's modified Eagle medium (DMEM) (Thermo Fisher) supplemented with 1% penicillin-streptomycin (Thermo Fisher), 1% L-glutamine (Thermo Fisher), and 10% fetal bovine serum (FBS) (Thermo Fisher). J-Lat 10.6 cell line, which was created by Dr. Eric Verdin (The Buck Institute for Research on Aging, Novato, CA, USA) Laboratory, was obtained from Dr. Robert F. Siliciano (Department of Medicine, Johns Hopkins University School of Medicine, Baltimore, MD, USA) and was cultured in RPMI 1640 (Thermo Fisher) supplemented with 1% penicillin-streptomycin (Thermo Fisher), 1% L-glutamine (Thermo Fisher), and 10% FBS (Thermo Fisher). The CD4^+^ T cells from healthy donors and HIV-1-infected individuals were cultured in RPMI 1640 supplemented with 1% penicillin-streptomycin, 1% L-glutamine, 10% FBS, 1/1,000 recombinant human interleukin 2 (IL-2) (R&D Systems). All cells had been tested and confirmed to be mycoplasma-free by PCR assays. All cells were maintained in a clean incubator at 37°C and 5% CO_2_.

### Reagents and antibodies

DMEM, FBS, the Lipofectamine 2000 reagents, 4′,6-diamidino-2-phenylindole (DAPI), and penicillin-streptomycin were obtained from Gibco. The TFD, MG132, and chloroquine were purchased from Selleck. The PUGNAc and GlcN were purchased from Thermo Fisher. The following antibodies were used for this study: anti-CHAF1A antibody (17037-1-AP, Proteintech), anti-OGT antibodies (11576-2-AP, Proteintech), anti-O-GlcNAc antibody (CTD110.6; #12938, CST), anti-HA antibody (M180-3, MBL), anti-DDDDK antibody (PM020, MBL), anti-GAPDH antibody (10494-1-AP, Proteintech), IRDye 680RD goat anti-mouse IgG antibody (926-68070, LI-COR Biosciences), IRDye 800CW goat anti-rabbit IgG antibody (926-32211, LI-COR Biosciences), and anti-rabbit IgG H&L (Alexa Fluor 488) (ab150077, Abcam).

### High-throughput screening

HEK293T cells (1 × 10^5^) were cultured in 200 µL of medium (DMEM with 10% FBS and 1% penicillin-streptomycin) in 96-well plates. The cells were co-transfected with 30 ng pEGFP-CHAF1A-C1 and 20 ng pcDNA3.1-CFP per well, in which fluorescence intensity of GFP was used to indicate degradation efficiency of drugs and CFP for quantification of cell viability (inversely correlated to compound toxicity). Four to six hours after transfection, the cells were treated with drugs from the “US DRUG COLLECTION" library at concentrations of 50 µM and dimethyl sulfoxide (DMSO) in the same volume as a negative control. Forty-eight hours later, the medium was thrown away, and the cells were washed in phosphate buffered saline (PBS), followed by detecting fluorescence intensity of GFP and CFP by Luminoskan Ascent Microplate Luminometers (Thermo Fisher). Then, the candidates of more than 50% degradation efficiency and less than 50% cell toxicity were calculated, analyzed, and picked up by normalizing with the negative control.

### Western blotting

HEK293T cells treated with TFD for 48 h were collected and lysed with NP40 lysis buffer (10 mM Tris-HCl buffered at pH 7.5, 150 mM NaCl, 0.5% NP-40, 1% Triton X-100, 10% glycerol, 2 mM EDTA, 1 mM NaF, 1 Mm Na3VO4) supplemented with protease inhibitor cocktail (Sigma Aldrich) on ice for 30 min. The lysis was vortexed every 10 min. Lysates were then clarified by centrifugation at 12,000 *g* for 5 min at 4°C, boiled at 100°C with loading buffer supplemented with DL-dithiothreitol (DTT) for 10 min and separated by SDS-PAGE. Proteins were transferred to nitrocellulose membranes (PALL). The membranes were then blocked with 5% non-fatty milk for 1 h at room temperature and incubated with anti-CHAF1A and anti-GAPDH antibodies overnight at 4°C. After three washes, membranes were incubated with IRDye secondary antibodies (LI-COR) for 1 h at room temperature and scanned with the Odyssey infrared imaging system (LI-COR).

### Co-IP assay

HEK293T cells were seeded on 6 cm plates and transfected with 3 µg CHAF1A-3HA and 4 µg 3Flag-ubiquitin. Transfection was performed using Lipofectamine 2000, according to the manufacturers’ instructions. Cells were harvested and lysed with 500 µL NP40 lysis buffer supplemented with 1/100 protease inhibitor cocktail (Sigma Aldrich) and 2 M N-Ethylmaleimide (NEM) (Selleck) on ice for 30 min. The lysis was vortexed every 10 min. Lysates were then clarified by centrifugation at 12,000 *g* for 5 min at 4°C, and 80  µL of the lysates was taken as input control. The remaining lysates were rotated with anti-HA beads for 4 h to overnight at 4°C. The beads were then washed three to five times with 1,000 µL ice-cold STN IP wash buffer (10 mM Tri-HCl buffered at pH 7.5, 150 mM NaCl, 0.5% NP-40, 0.5% Triton X-100). Proteins were eluted with loading buffer supplemented with DTT at 100°C for 10 min. The CHAF1A ubiquitination was detected by western blotting using anti-HA (M180-3, MBL), anti-Flag (PM020, MBL), and anti-GAPDH (10494-1-AP, Proteintech) antibodies.

### Immunofluorescence assays

HEK293T cells were seeded on chambered coverglass (Thermo Fisher) and treated with TFD. Forty-eight hours later, cells were washed three times with PBS and fixed with 4% paraformaldehyde at room temperature for 10 min. The cells were washed three times with PBS and blocked with PBS containing 0.5% bovine serum albumin (BSA) at room temperature for half an hour. Cells then were incubated with anti-CHAF1A antibody (17037-1-AP, Proteintech) at 4°C overnight. Following washing with 0.1% Tween 20-PBS (PBS-T) three times, the cells were incubated with goat anti-rabbit IgG H&L (Alexa Fluor 488) (ab150077, Abcam) at room temperature for an hour. Cells then were washed with 0.1% PBS-T three times and incubated with DAPI at room temperature for 5 min, after which the cells were washed with 0.1% PBS-T three times. Fluorescent images were captured and analyzed with the N-SIM module of the NIS-Elements Advanced Research software (Nikon).

### Luciferase assay

TZM-bl cells treated with TFD for 48 h were collected and lysed with passive lysis buffer (Promega) for 30 min at room temperature. Lysates were then clarified, and luciferase in the cell lysates was measured with a luciferase reporter assay system (Promega).

### qPCR

The identity of whether the TFD influences CHAF1A mRNA, HEK293T Cells treated with TFD for 48 h were collected and lysed with TRIzol reagent (Thermo Fisher) and proceeded to cDNA synthesis with PrimeScript RT reagent Kit (Takara). Quantitative PCR (qPCR) was conducted using SYBR EX-Taq Premix (TaKaRa) in a CFX96 real-time PCR detection instrument (Bio-Rad). The data were analyzed by a SYBR green-based system (Bio-Rad) and normalized to GAPDH. Primer pairs were the following: *GAPDH*, forward, 5′ ATC CCA TCA CCA TCT TCC AGG 3′; reverse, 5′ CCT TCT CCA TGG TGG TGA AGA C 3′; *CHAF1A*, forward, 5′ TTA GAC CGA AAC TTG TCA ACG G 3′; reverse, 5′ GTC TGG CTG CTC ATT CGA GT 3′. The relative expression of each gene was calculated as 2^[Ct(Control-TRIM28)-Ct(Control-b-Actin)]-[Ct(Treatment-TRIM28)-Ct(Treatment-b-Actin)]^. For the quantification of HIV-1 reactivation in primary CD4^+^ T cells of HIV-1-infected individuals, the procedure was previously described (31–33). Briefly, qPCR was performed for specific reverse-transcribed HIV-1 cDNA with primer pairs: HIV-TotRNA Forward Primer: 5′-CTGGCTAACTAGGGAACCCACTGCT-3′ and HIV-TotRNA Reverse Primer: 5′-GCTTCAGCAAGCCGAGTCCTGCGTC-3′ (31). After quantitation, an *in vitro*-transcribed HIV-1 RNA was used as the external control for measuring cell-associated viral RNAs. The Ct of each group was converted to mass and further converted to copies. The final expression of intracellular HIV-1 RNA was represented as 10^3^ copies of viral RNA per million CD4^+^ T cells.

### siRNA transfection

HEK293T cells were transfected with 100 nM of negative control, OGT-specific siRNA (siOGT-1: 5′- GGCACAAACTTCCGAGTGA −3′, siOGT-2: 5′- GCAGAAGCTTATTCGAATT −3′, siOGT-3: 5′- GCAGTTCGCTTGTATCGTA −3′) (Ribobio), or OGA-specific siRNA (siOGA-1: 5′- GCAGTTACTTGCTGATCTA-3′, siOGA-2: 5′- GCAAGAAGATTGTATTAGT-3′, siOGA-3: 5′- GGCACTTTCTGTTATCCAA-3′) (Ribobio), with Lipofectamine 2000 (Invitrogen) according to the manufacturer’s instructions. Forty-eight hours later, the cells were collected and lysed for further western blot.

### shRNA-mediated knockdown and flow cytometry

The shRNA target sequence against OGT CDS was 5′ GCTGAGCAGTATTCCGAGAAA 3′. The shRNA targeting luciferase (shluc: 5′-ACCGCCTGAAGTCTCTGATTAA-3′) was set as the negative control (34). Target sequences were cloned into pLKO.3G-RFP which was derived from pLKO.3G, in which the GFP-tag was replaced with RFP-tag in pLKO.3G-RFP. pLKO.3G-RFP-shOGT and pLKO.3G-RFP-shLUC were produced in HEK293T cells by co-transfecting 3 mg of VSV-G (Addgene plasmid # 12259) glycoprotein-expression vector, 6 mg of lentiviral packaging construct pCMVDR8.2, which was a kind gift from Dr. Didier Trono (School of Life Sciences, Ecole Polytechnique Fédérale de Lausanne, Lausanne, Switzerland) (35), and 6 µg shRNA expression lentiviral construct using Lipofectamine 2000 (Thermo Fisher) according to the manufacturer’s instruction. The yield virus was concentrated into 1 mL RPMI 1640 by PEG 6000. J-Lat10.6 cells were spin-infected with shRNA virus. Forty-eight hours later, infected cells were harvested to identify knockdown efficiency by western blot, detecting infection efficiency by measuring the percentage of RFP-positive cells and confirming the reactivation effect by testing the GFP-positive cells using flow cytometry. The fluorescence value was analyzed by FlowJo software.

### Cell viability assay

The cell viability assay was conducted by measuring the percentage of amine-reactive fluorescent dye (BioLegend, 423113) non-permeant cells by flow cytometry, which indicated the percentage of viable cells.

### TZM-bl qVOA

Peripheral blood and rectal tissue were collected from ART-treated HIV-infected individuals. Rectal tissue was digested in DPBS (containing Ca²^+^/Mg²^+^) supplemented with 1.0 mg/mL Collagenase IV, 1.5 mg/mL Dispase II, 0.5 mg/mL Hyaluronidase, 30 µg/ml DNase I, 1% BSA, and 1% penicillin/streptomycin at 37°C for 30–60 min. The tissue was gently pipetted to obtain a single-cell suspension, which was then filtered through a 70 µm cell strainer. PBMCs were isolated by Ficoll density gradient centrifugation after 1:2 dilution with PBS. All cells were washed twice with PBS and resuspended in culture medium. PBMCs or rectal cells were seeded at 1 × 10^5^ to 2 × 10^5^ per well in 24-well plates and treated with TFD (final concentrations 50 and 100 µM) and Dynabeads CD3/CD28 (12.5 µL per 1 million cells). Cells were cultured for 5 days in IMDM or RPMI 1640 with 10% FBS and 1% penicillin-streptomycin, with IL-2 supplementation. Controls received vehicle only. On day 5, activated cells were collected, counted, and washed twice with PBS. TZM-bl cells were seeded at 5 × 10^4^ per well in 96-well white plates one day in advance. Activated T cells were added onto TZM-bl cells and co-cultured for 48–72 h. After co-culture, gently remove supernatants and wash wells twice with PBS. Add 100 µL Bio-Lite Luciferase Assay Working Solution to each well, incubate at room temperature (25°C) for 5 min in the dark, and immediately measure luminescence using a plate reader.

### sWGA pull-down assay

Cells were lysed in ice-cold lysis buffer (50 mM Tris-HCl, pH 7.4, 150 mM NaCl, 1% NP-40, 1 mM EDTA, and protease inhibitor cocktail) for 30 min on ice. Cell lysates were centrifuged at 12,000 × *g* for 15 min at 4°C to remove debris. The supernatants were collected, and protein concentrations were determined using the BCA assay (Thermo Fisher Scientific). For each sample, 250 µg total protein was incubated overnight at 4°C with 50 µL of pre-washed succinylated wheat germ agglutinin (sWGA) agarose beads (Vector Laboratories) to enrich O-GlcNAcylated proteins. After incubation, the beads were washed three times with lysis buffer containing 0.1% Tween-20 and twice with TBS to remove non-specifically bound proteins. Bound proteins were eluted by boiling in 60 µL of 3 × SDS sample buffer for 10 min at 95°C. The input and sWGA-enriched samples were analyzed by SDS-PAGE and immunoblotting with antibodies anti-O-GlcNAc (CTD110.6), anti-CHAF1A (17037-1-AP, Proteintech), and anti-GAPDH(10494-1-AP, Proteintech).

### Rescue experiments with CHAF1A overexpression

TZMBL cells were seeded in 24-well plates and first transfected with Tat expression plasmid to activate luciferase expression. After Tat transfection, cells were treated with DMSO or 20 µM TFD for a specified period. Subsequently, different amounts of CHAF1A plasmid (CHAF1A-1μg and CHAF1A-2μg) were transfected into designated wells. After incubation, single-luciferase reporter activity was measured using a luciferase assay kit according to the manufacturer’s instructions. All experiments were performed in triplicate. Statistical differences between groups were analyzed by one-way analysis of variance (ANOVA) followed by *post hoc* tests.

### CRISPR-Cas9-mediated sgRNA targeting OGA/sgRNA targeting OGT knockout in J-Lat10.6 cells

sgRNA sequences targeting OGA (5′-CTTTGGGTCCATGCTCGTA-3′) and OGT (5′-GCACGCGTATAACACTGCA-3′) genes were designed using the respective gene sequences. For cloning into the lentiCRISPR v2 vector (Addgene #52961), BsmBI restriction site-compatible overhangs were added as follows:

OGA forward: 5′-caccgCTTTGGGTCCATGCTCGTA-3′

OGA reverse: 5′-aaacTACGAGCATGGACCCAAAGc-3′

OGT forward: 5′-caccgGCACGCGTATAACACTGCA-3′

OGT reverse: 5′-aaacTGCAGTGTTATACGCGTGCc-3′

All oligonucleotides were synthesized by Ruibo Biotech (Guangzhou, China). The forward and reverse oligos were annealed and ligated into BsmBI-digested lentiCRISPR v2. Recombinant plasmids were verified by colony PCR and Sanger sequencing. Oligos were synthesized with BsmBI-compatible overhangs, annealed, and ligated into BsmBI-digested lentiCRISPR v2 vector. Recombinant plasmids were confirmed by colony PCR and Sanger sequencing. Lentiviral packaging was performed by co-transfecting HEK293T cells with lentiCRISPR v2-sgRNA, VSV-G, and psPAX2, followed by viral concentration. J-Lat10.6 cells were infected with concentrated virus, and after 48 h, cells were selected with puromycin at the optimal concentration determined by a kill curve. Knockout efficiency was verified by western blot.

### Statistical analysis

The results of the experiments are presented as means ± standard errors of the means (SEM). Student’s unpaired *t*-test was used to determine significance. *P* values are denoted in figures as ns: no significance, *: *P* < 0.05, **: *P* < 0.01, and ***: *P* < 0.001.

### Data analysis

Bulk RNA sequencing data were obtained from the Gene Expression Omnibus (GEO) database under accession numbers GSE166337 and GSE178670. Mass spectrometry proteomics data were retrieved from ProteomeXchange Consortium (PXD024172). Raw RNA-seq counts were filtered and analyzed using the DESeq2 package (44). Gene expression comparison between groups used normalized counts from DESeq2’s normalization method, with statistical significance assessed by Wilcoxon test. For integrative bioinformatics analysis, both transcriptomic and proteomic data were subjected to Gene Ontology (GO) and KEGG pathway enrichment analysis using clusterProfiler package (45). Gene Set Enrichment Analysis (GSEA) was performed based on the pre-ranked gene lists to identify significantly enriched pathways. The correlation between genes was evaluated using Pearson correlation analysis.

## Data Availability

The original data and materials generated in this study are publicly accessible. Mass spectrometry-based proteomics data have been deposited in the ProteomeXchange Consortium via PRIDE under data set identifier PXD024172. RNA-seq data are available in the NCBI GEO under accession number GSE166337, GSE166337, and GSE178670. Newly generated RNA-seq data from TFD-treated J-Lat 10.6 cells have been deposited in the Sequence Read Archive (SRA) under BioProject accession number PRJNA1304135. All full Western blot images for our figures and high-throughput sequencing (HTS) data have been deposited in the Zenodo digital repository (https://doi.org/10.5281/zenodo.17453809) and are publicly accessible.

## SUPPLEMENTAL MATERIAL

Supplemental figures jvi.01518-25-s0001.pdf Figures S1 to S6.

ASM does not own the copyrights to Supplemental Material that may be linked to, or accessed through, an article. The authors have granted ASM a non-exclusive, world-wide license to publish the Supplemental Material files. Please contact the corresponding author directly for reuse.
