# Supplementary material for: The crosstalk between ubiquitination and GlcNAcylation of CHAF1A regulates HIV-1 latency and reactivation
Source: J Virol. 2025 Dec 3;99(12):e01518-25. doi: 10.1128/jvi.01518-25 (PMC12724330; doi:10.1128/jvi.01518-25)
Supplement: Supplemental figures — Figures S1 to S6. [file jvi.01518-25-s0001.pdf]

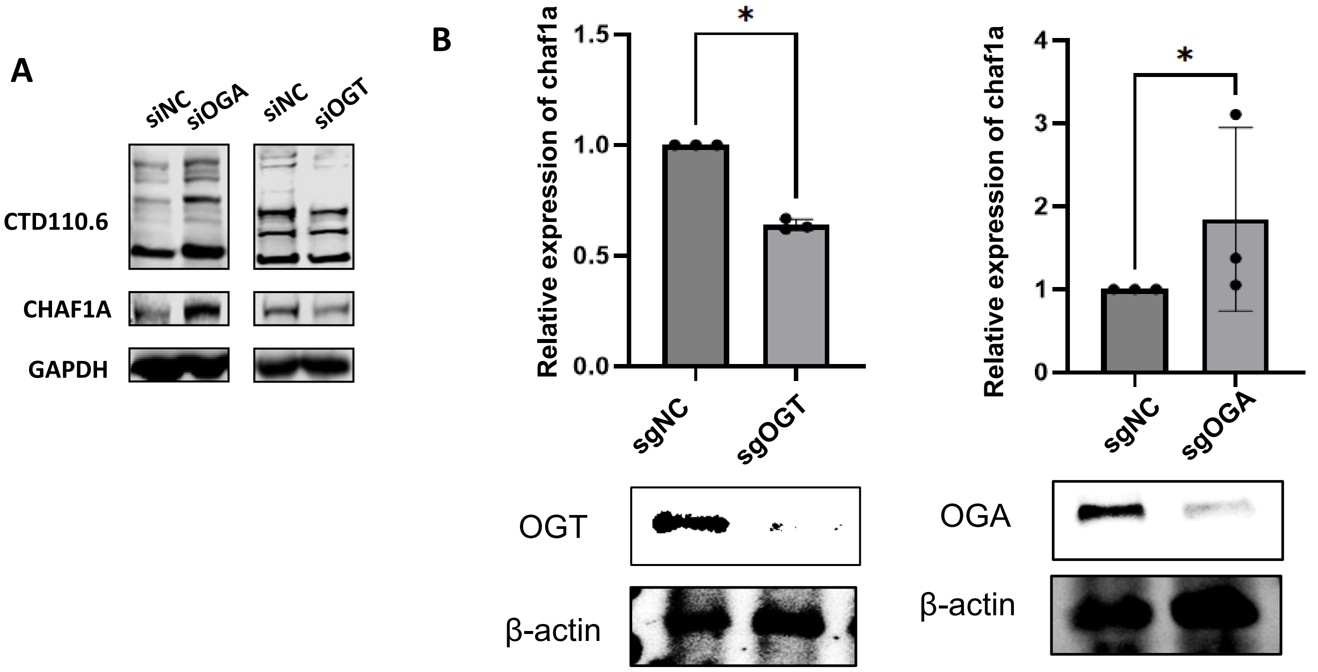

Figure S1. Effects of OGT and OGA knockdown on CHAF1A expression.

(A) siRNA-mediated knockdown of OGT and OGA, followed by Western blot analysis of endogenous CHAF1A. Knockdown efficiency and CHAF1A expression levels were assessed by Western blot using anti-O-GlcNAc and anti-CHAF1A antibodies. GAPDH served as a loading control.

(B) Quantification of CHAF1A protein levels following knockdown of OGT (sgOGT) or OGA (sgOGA) relative to sgNC, based on densitometric analysis of three independent Western blots. *$P < 0.05$ by Mann–Whitney test.

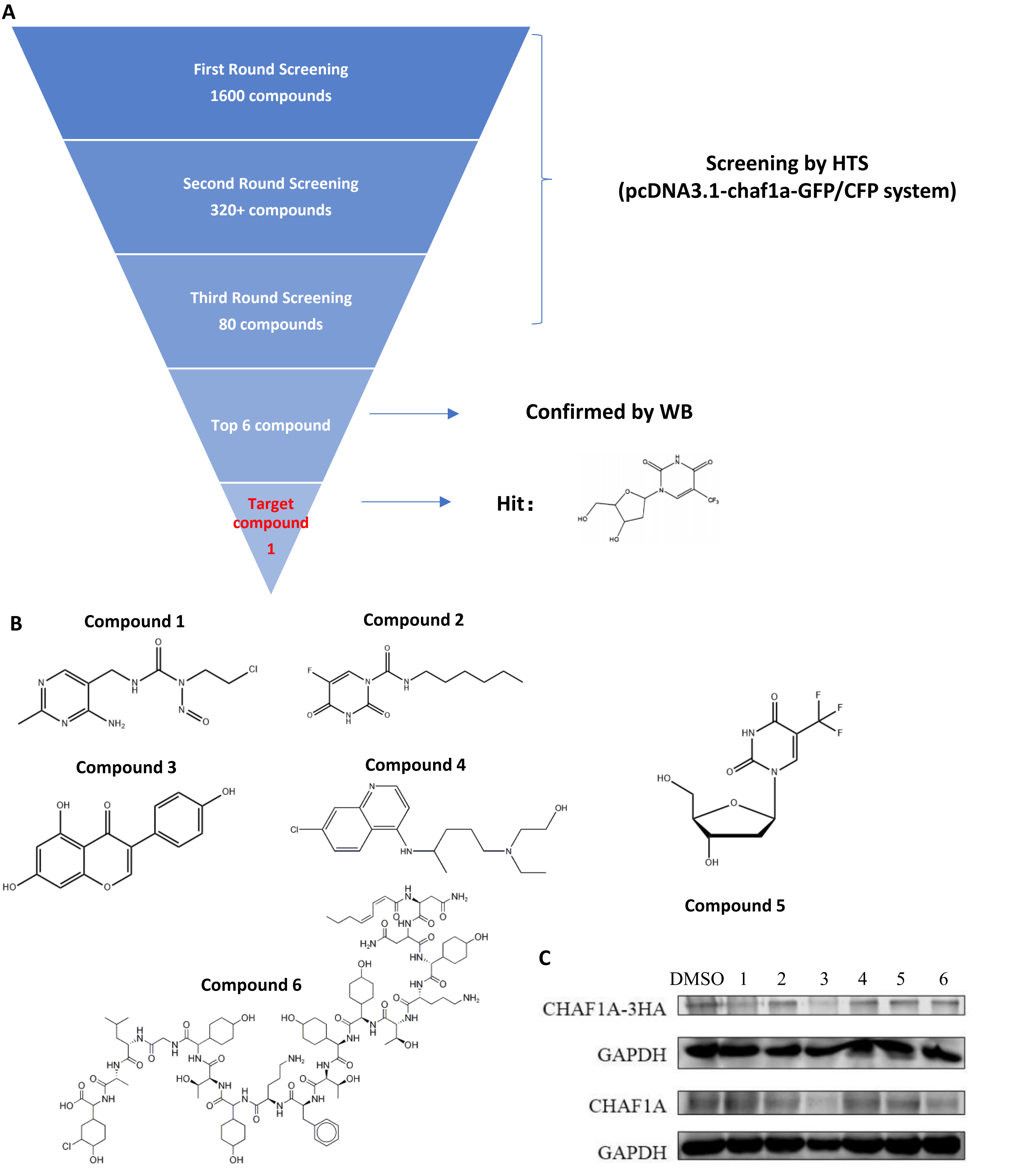

Figure S2. High-throughput screening workflow.

(A) Hit compound identification.

(B) Chemical structures of the six identified hits.

(C) HEK293T cells were treated with 20 μM of various compounds for 48 hours, followed by cell harvest and analysis via Western blotting using a CHAF1A-specific antibody.

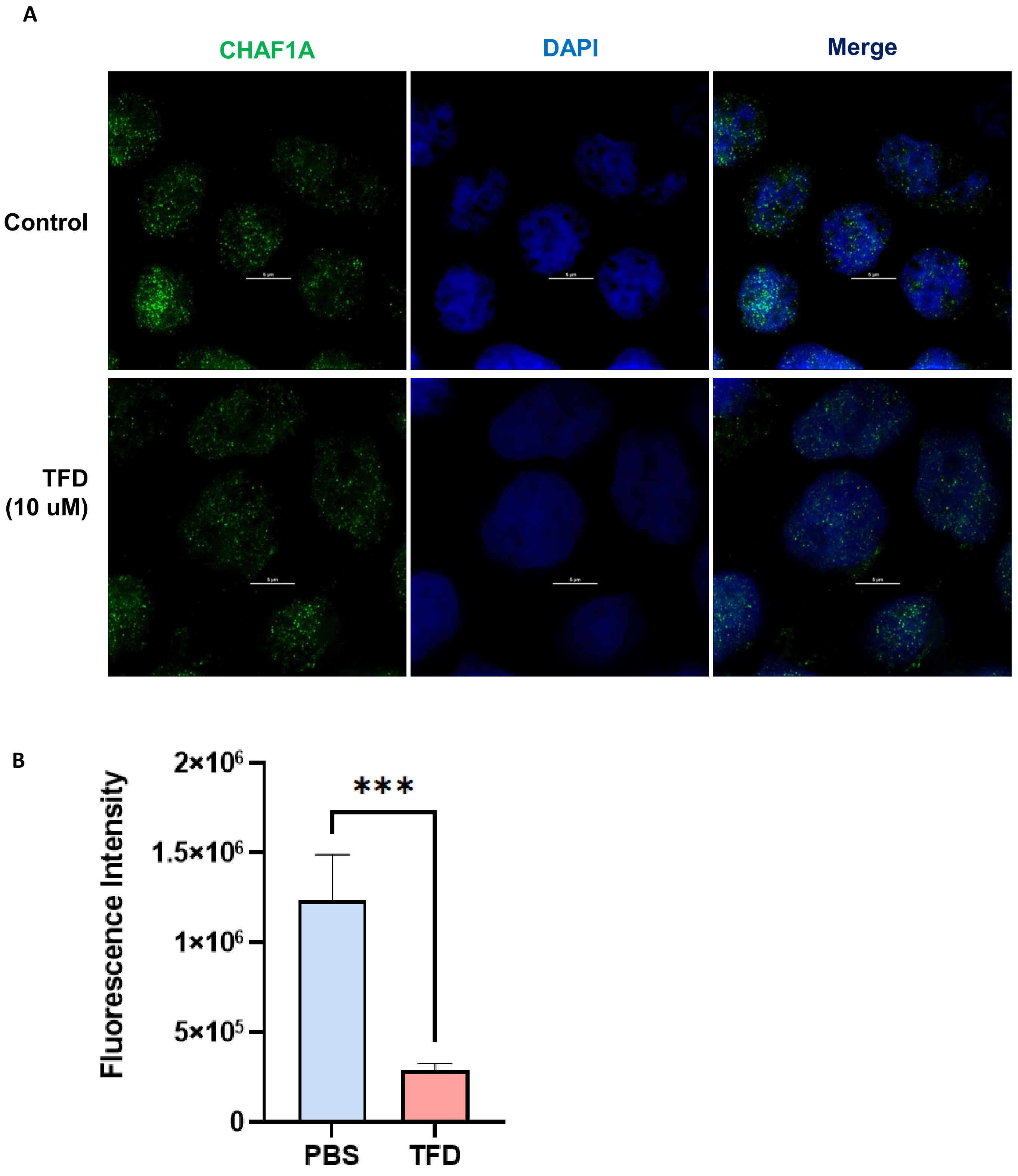

Figure S3. TFD treatment reduces CHAF1A protein levels in cells.

(A) Representative immunofluorescence images show CHAF1A (green) and DAPI (blue) staining in cells treated with PBS (control) or TFD (10 μM) for 48 hours. Scale bar: 5 μm. Merged images depict the overlay of CHAF1A and DAPI signals.

(B) Quantification of CHAF1A fluorescence intensity is presented in Fig. S3A. Data are expressed as mean ± SD; ***$P < 0.001$ by unpaired t-test.

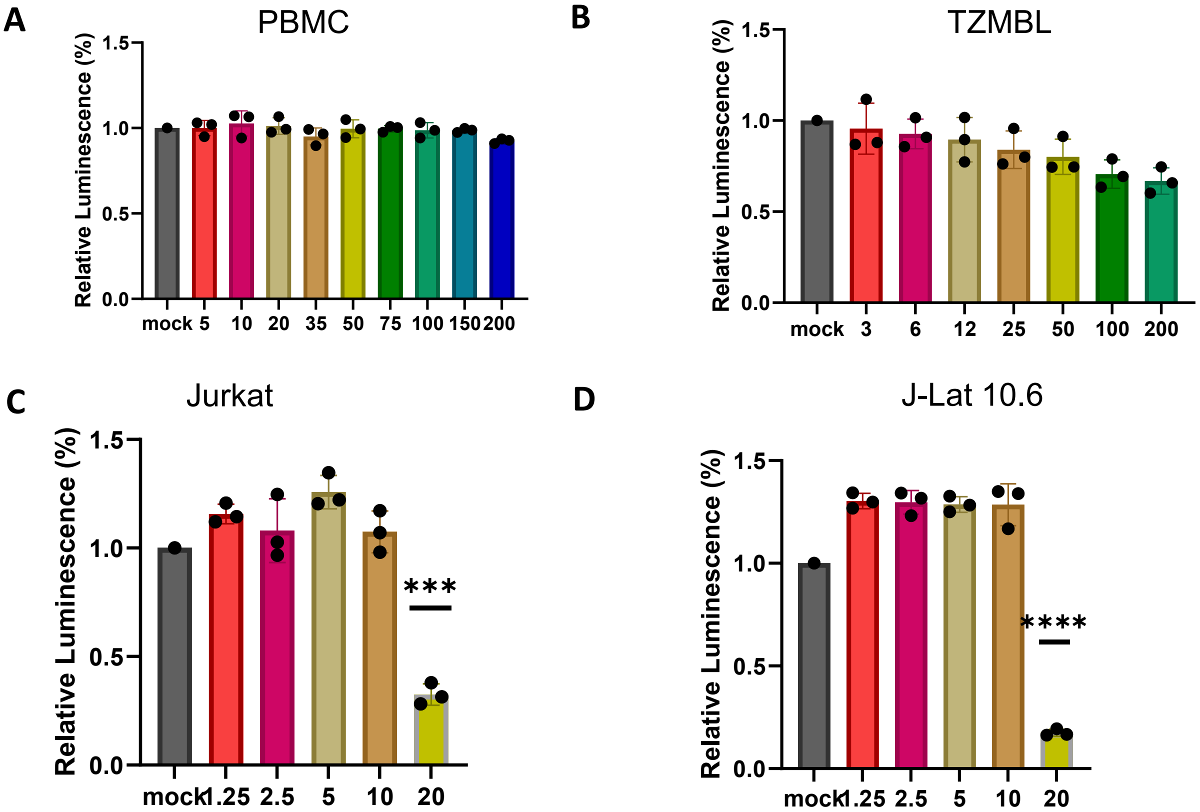

Figure S4. Dose-dependent effects of TFD on cell viability across different cell types.

Cell viability was assessed using the CellTiter-Glo assay following a 48-hour treatment with TFD. (A) PBMCs and (B) TZM-bl cells maintained >80% viability at all tested concentrations. Jurkat (C) and J-Lat 10.6 (D) exhibited dose-dependent cytotoxicity, with significant cell death observed at 20 μM. Data represent mean ± SEM from three independent experiments. Statistical analysis was performed using one-way ANOVA with Dunnett's test.

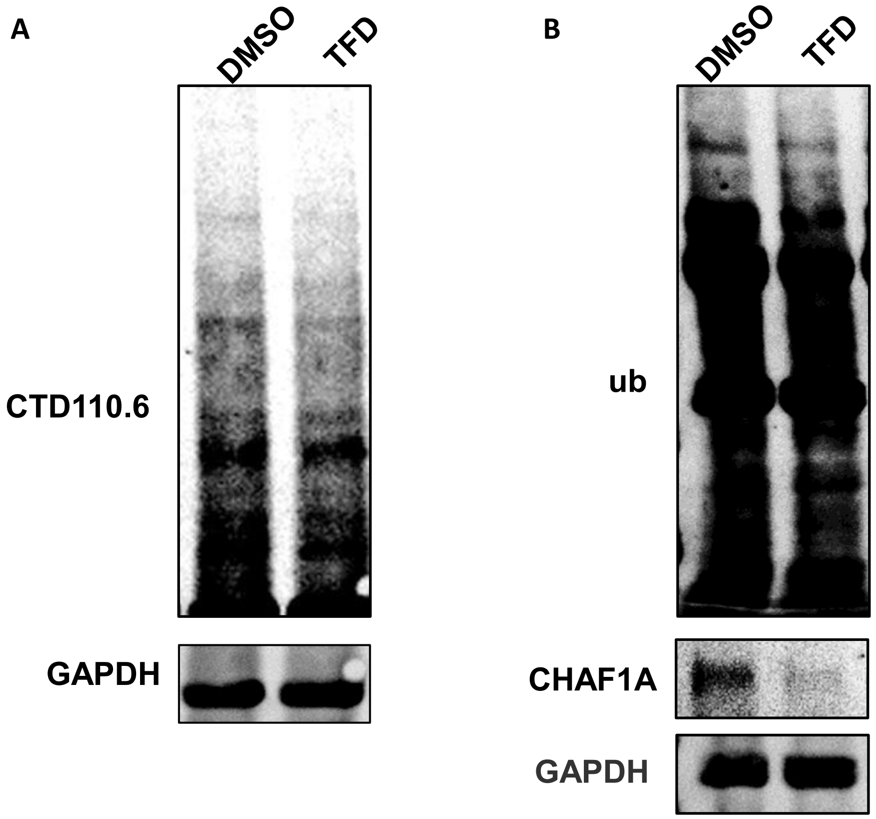

Figure S5. TFD reduces global O-GlcNAcylation levels in primary CD4^{+} T cells.

(A) Representative immunoblot analysis of O-GlcNAcylated proteins in human primary CD4^{+} T cells were treated with DMSO or 10 μM TFD. O-GlcNAcylation was detected using the CTD110.6 antibody. GAPDH served as a loading control.

(B) Primary CD4^{+} T cells were activated with anti-CD3/CD28 beads for 2 days, followed by treatment with 10 μM trifluridine (TFD) or DMSO (control) for an additional 2 days. Cell lysates were analyzed by immunoblotting with the ub and chaf1a antibodies. GAPDH was used as a loading control.

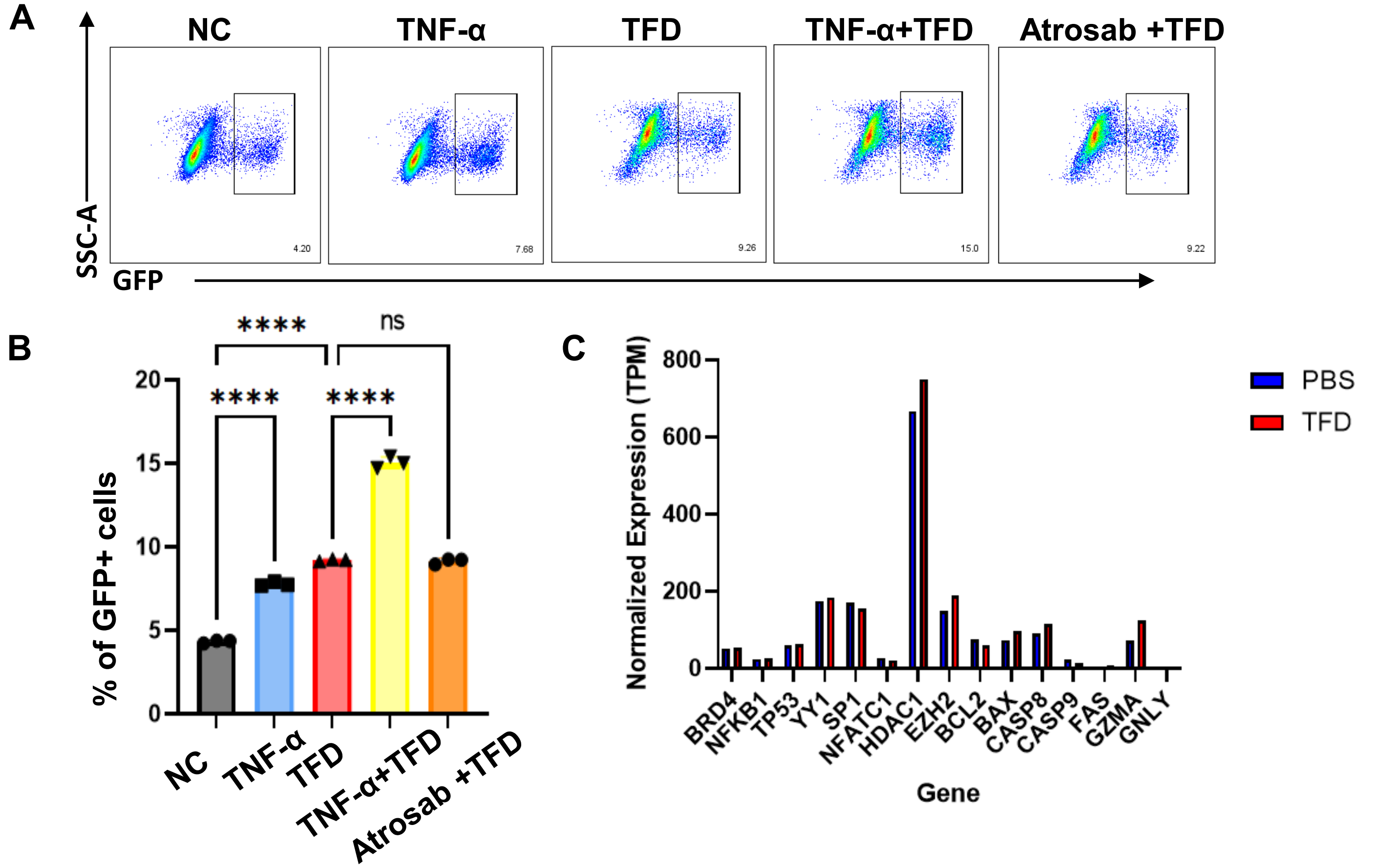

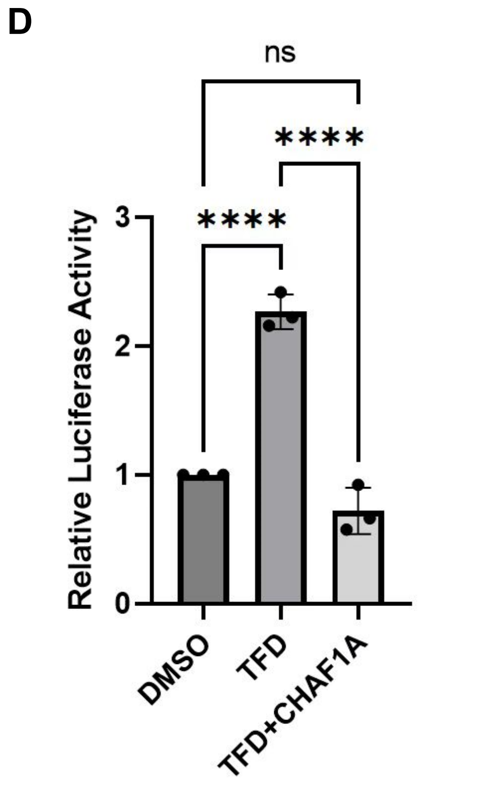

Figure S6. TFD and TNF-α synergistically reactivate HIV-1 latency, whereas TFD-mediated reactivation is independent of TNF-α receptor signaling.

(A) Representative flow cytometry plots showing the percentage of GFP$^+$ cells under the following treatment conditions: NC (negative control), TNF-α (5 ng/mL, 2 days), TFD (2 μM, 2 days), TNF-α + TFD (5 ng/mL TNF-α and 2 μM TFD, 2 days), and Atrosab + TFD (pretreatment with Atrosab at 40 μg/mL for 1 day, followed by 2 μM TFD for 2 days).

(B) Quantification of GFP$^+$ cell percentages from three independent experiments. Data are presented as mean ± SD; statistical analysis was performed using ordinary one-way ANOVA. ****$P < 0.0001$, ns: not significant.

(C) J-Lat 10.6 cells were treated with 10 μM TFD, with DMSO serving as a control. After 48 hours, cells were harvested and RNA was extracted for RNA sequencing. Normalized mRNA expression levels (in TPM) of key HIV latency-associated genes in cells treated with PBS (blue) or TFD (red) are shown.

(D) TZM-bl cells were treated with DMSO, TFD, or TFD in combination with CHAF1A plasmid transfection. Relative luciferase activity was measured and is presented as mean ± SD ($n = 3$). Statistical significance was determined by one-way ANOVA with post hoc tests; ns, not significant; *$p < 0.05$; **$p < 0.01$; ***$p < 0.001$.
